# Spontaneous Preterm Delivery, Particularly with Reduced Fetal Growth, is Associated with DNA Hypomethylation of Tumor Related Genes

**DOI:** 10.4172/2376-127X.1000215

**Published:** 2016-01-29

**Authors:** Xinhua Chen, Guang Bai, Theresa O Scholl

**Affiliations:** 1 Department of Obstetrics and Gynecology, Rowan University - School of Osteopathic Medicine, Stratford, NJ, USA; 2 Department of Neural and Pain Sciences, University of Maryland, School of Dentistry, Baltimore, MD, USA

**Keywords:** Spontaneous preterm delivery, Reduced fetal growth, DNA methylation, Tumor related genes, Free choline, Total choline

## Abstract

**Background:**

Preterm delivery and sub-optimal fetal growth are associated with each other and affect both mother and infant. Our aim was to determine (i) whether there are detectable differences in DNA methylation between early and late gestation and (ii) whether changes in DNA methylation from entry are associated with spontaneous preterm delivery with and without reduced fetal growth.

**Methods:**

We conducted a case-control study nested within a large prospective cohort. Gene specific methylation was measured by Methyl-Profiler PCR Array in a Human Breast Cancer Signature Panel of 24 genes from maternal peripheral leukocytes genomic DNA at entry and 3^rd^ trimester (sampled at 16 and 30 weeks of gestation, respectively). Clonal bisulfite DNA sequencing was performed to confirm the changes in selected genes (*CYP1B1, GADD45A and CXCL12*). Multivariable analysis was used for data analysis.

**Results:**

There was significantly decrease in DNA methylation in 15 of 24 genes during the 3^rd^ trimester in cases of spontaneous preterm delivery (n=23) as compared to the controls (n=19) (p<0.05–p<0.01 for each gene). Similar results were observed by bisulfite sequencing for 3 genes. The change in DNA methylation between late and early gestation was significantly different in cases (overall decrease in methylation was −4.0 ± 1.5%) compared to the controls (overall increase in methylation was 12.6 ± 2.19%, p<0.0001). A graded pattern of DNA methylation was observed in 15 genes. Cases who delivered preterm with reduced fetal growth had the lowest level of methylation, cases delivering preterm without reduced fetal growth were next and term controls were highest in methylation (p for trend <0.05 to p<0.01 for each gene). Cases of preterm delivery also had significantly lower dietary choline intake.

**Conclusions:**

These data suggest that epigenetic modification is associated with an increased risk of spontaneous preterm delivery, spontaneous preterm delivery with reduced fetal growth in particular.

## Introduction

Preterm delivery complicates 10–12% of all US births and is a serious health problem for both mother and infant [1–4]. Infants delivered preterm are also at an increased risk of low birth weight (LBW, <2500 g), being small-for-gestational age (SGA), or otherwise reduced in their utero growth when compared to infants delivered at term [2,5]. While SGA is a common reason for an indicated preterm delivery, reduced fetal growth is also associated with an increased risk of spontaneous preterm delivery [2,6]. Apart from medically indicated preterm delivery, the underlying causes and mechanisms of spontaneous preterm delivery remain largely unknown [7].

DNA methylation is the best understood mechanism and the most common marker used in epigenetic studies [8]. Alternation in DNA methylation of certain genes plays an important role in many pathological conditions that modify disease risk during pregnancy [9,10] as well as in the non-pregnant state [11,12]. Epigenetic changes influence several pregnancy outcomes including preterm birth, the duration of gestation and the growth trajectory of the fetus, infant and child [13–15]. In most cases specimens are often, but not exclusively, neonatal and obtained at delivery [9,14–16]. Prospective data on changes in maternal DNA methylation during pregnancy and their influence on fetal growth and gestation in human pregnancy are both limited and inconsistent [9,14,16].

Cancer and pregnancy have many parallels including the need to establish a nutrient supply for tissue growth and differentiation and to evade immune surveillance [17]. Recent reports suggested that some tumor-related genes are expressed during pregnancy [18–21]. For example, pre-eclampsia associated stressors (the inflammatory cytokines IL-6 and TNF-α, hypoxia and angiotensin II) induce expression of Growth Arrest and DNA Damage-inducible 45 genes (*GADD45*a) in human placental explants [18]. Cord blood DNA methylation of tumor genes (*CDKN1C, EPHA1, MPL*) is associated with childhood body size [19]. In animal models, some cancer related genes (e.g., transformation related protein 53 (*p53*) have multiple functions (oxidative stress, inflammation) that can alter the outcome of pregnancy (e.g., preterm birth) [20,21]. Thus, we analyzed DNA methylation in 24 genes from a Human Breast Cancer Signature Panel. Many genes in this panel influence the course of pregnancy; their dysfunctional expression or mutation is related to fetoplacental abnormalities during gestation (see S1 Table). We used maternal peripheral leukocytes DNA to examine (i) Whether there are detectable differences in DNA methylation between early and late gestation; (ii) Whether changes in DNA methylation from entry are associated with spontaneous preterm delivery with and without reduced fetal growth and (iii) Whether dietary nutrient intake including folate and choline are different between cases and controls.

## Materials and Methods

### Study design and subjects

We conducted a case-control study nested within the Camden Study, a large prospective epidemiological study of young, generally healthy pregnant women residing in one of the poorest cities in the continental United States [22]. The underlying cohort of study participants enrolled between 1996 and 2006 were recruited from among patients enrolling at the Osborn Family Health Center, Our Lady of Lourdes Medical Center and St John the Baptist prenatal clinic in Camden, NJ. The study protocol was approved by the institutional review board at the University of Medicine and Dentistry of New Jersey (which later became Rowan University - School of Osteopathic Medicine in 2013). Informed written consent was obtained from each participant at enrollment after explanation of the nature and purpose of the study.

A total of 3.5% of the women who had serious non-obstetric problems (e.g., Lupus, type 1 or 2 diabetes, seizure disorders, malignancies, acute or chronic liver disease, drug or alcohol abuse and psychiatric problems) were not eligible. Eighty percent of the patients who were eligible agreed to participate. A total of 8.3% of participants dropped out after enrollment due either to a move from the area or to an early pregnancy loss. A final total of 2,379 participants whose pregnancy culminated in a live birth were used to randomly select cases of spontaneous preterm delivery and controls whose infants were delivered at term.

### Data and blood samples collection

Data on socioeconomic, demographic, lifestyle and anthropometric variables were obtained at entry to prenatal care (13.5 ± 0.7 weeks of gestation), and updated at gestational weeks 20 and 28. Ethnicity was self-defined. Blood samples were collected at entry (16.1 ± 0.7 gestational weeks, mean ± SE) and during the 3^rd^ trimester (29.5 ± 0.5). A standardized protocol for biological specimen collection is used for the Camden Study. Briefly, whole venous blood was collected into EDTA - containing Vacutainer tubes (Becton, Dickinson and Company, Franklin Lakes, NJ) and centrifuged to allow for collection of plasma and the buffy coat. The buffy coat enriched in white blood cells was used for DNA extraction. All buffy coats were prepared under optimal conditions within 2 hr after blood collection and immediately aliquoted and stored at −80°C and not thawed until analyzed.

### Dietary data

A 24-hour recall of the previous day’s diet was obtained at entry to care, week 20 and 28 gestation processed with databases from the Campbell Institute of Research and Technology (Campbell Soup Company) in Camden as described previously [23]. The database generates data for more than 70 nutrients including choline (free and total) intake using the United States Department of Agriculture (USDA) Nutrient Database for Standard Reference (http://www.nal.usda.gov/fnic/foodcomp), the Continuing Survey of Food Intakes by Individuals, and USDA database for the choline contents of common foods (release two, 2008) (http://www.ars.usda.gov/sp2UserFiles/place/80400525/Data/choline/cholh01.pdf).

### Definition of cases and controls

Preterm delivery is defined as delivery at <37 completed weeks of gestation based upon the last menstrual period confirmed or modified by ultrasound evaluation [24]. Detailed information identifying women with spontaneous preterm delivery, and medically indicated preterm delivery as well as fetal growth measures including infant birth weight, length, the circumferences of head and chest were obtained from the prenatal, labor, deliver and newborn records. Information on reproductive history including prior preterm delivery as well as the medical events during the current pregnancy was also obtained by interview and/or abstracted from clinical records.

Total preterm delivery was 10.1% in the underlying cohort (cases=240). Spontaneous preterm delivery was defined by the presence of intact membranes and regular contractions and by the absence of induction of labor or an elective caesarean section. Preterm premature rupture of membrane (PROM) was defined as rupture of membranes before the onset of labor in the spontaneous preterm group. Women with medically indicated preterm delivery (20% of total preterm), those with a multiple pregnancy and/or with a prior history of preterm delivery were excluded from sample selection. Cases of spontaneous preterm delivery (n=23) and term controls (n=19) were randomly selected by SAS PROC SURVEYSELECT (SAS Institute, Inc., Cary, NC) to assure that distribution of maternal characteristics was similar in cases and controls.

Infant weight below the 25^th^ percentile for gestational age was defined as reduced fetal growth using a standard that adjusted for maternal parity, ethnicity and infant gender [25,26].

### Analytic procedures for DNA methylation and bisulfite sequencing

Genomic DNA (gDNA) was extracted from buffy coat of blood using Blood & Cell Culture DNA kit according to manufacturer’s instruction (Qiagen, Frederick, Maryland), quantified by NanoDrop 2000 Spectrophotometer (Thermo Scientific, Wilmington, DE) and examined on 0.7% agarose gel electrophoresis for DNA integrity. Samples showing an A260/A280 ratio >1.7, but <1.9, and a major band around 30 kb were used. Gene specific methylation was measured by Methyl-Profiler PCR Array in a Human Breast Cancer Signature Panel of 24 genes (see detailed information in S1 Table) (Catalogue MeAH012A, Qiagen). The assay is fast and provides accurate detection of DNA methylation status at selected CpG islands. It is based on the MethylScreen method of combined digestion of methylation-sensitive type II enzyme (HpaII/HhaI) and methylation-dependent type IV enzyme (McrBC) coupled to real-time PCR analysis of post-digested gDNA and was performed in triplicate as described previously [27,28]. Primers were designed, evaluated and provided by Qiagen.

The data processing was completed by web-based software provided by the manufacturer (Qiagen). Cycle threshold (Ct) values for each condition were used to calculate un-methylated (UM), fully methylated (FM) and intermediately methylated (IM) DNA such that UM, FM and IM sum to 1.0 for a given sample. The final results were the proportion (%) of methylated (sum of FM and IM) DNA to the total of unmethylated DNA (UM). All experiments were conducted blind without knowledge of case control status.

We selected three genes (*CYP1B1, GADD45A* and *CXCL12*) which showed significant differences by Methyl-Profiler PCR Array to confirm DNA methylation using sodium bisulfite modification followed by PCR-cloning-DNA sequencing [29]. Briefly, maternal gDNA (0.3 μg) were treated by sodium bisulfite using EZ DNA methylation kit (Zymo Research, Irvine, CA). Bisulfite treatment of denatured DNA converts all unmethylated cytosines to uracils, leaving methylated cytosines unchanged, and allowing for quantitative measurement of cytosine methylation status [30]. Three genes above were amplified by PCR with primers designed for modified gDNA using MethylPrimer software [31] and the following primer sets were used:

**Table:** 

Gene	Primer	seq (5′ --> 3′)
*CXCL12*	Upstream	gttttttattggtttttatttagtttt
	Downstream	acctttaaccttctcaaactcc
*CYP1B1*	Upstream	tttgggttgaggaaggtgtt
	Downstream	caaccaaccaaccttcacct
*GADD45A*	Upstream	ggttgagggttggtaggataatt
	Downstream	cctactttctacactcactcacaaac

### Statistical analysis

Maternal characteristics between cases and controls were compared by Student’s t tests (for continuous variables) and x2 tests or Fisher’s exact test (for categorical variables). Multivariate analysis of variance (MANOVA) was used to assess the significance of linear trend and compare mean levels of DNA methylation among cases and controls after the adjustment for potential confounding variables. The same method was used to test differences among subgroups of the cases - women who delivered a preterm infant with or without reduced fetal growth versus controls. The Bonferroni procedure was used to correct for multiple comparisons. The general linear model procedure (SAS, Proc GLM) was used for MANOVA. A class variable was coded for all cases, or cases with and without reduced fetal growth and controls. The dependent variable was DNA methylation (%) for each gene examined. All multivariable adjusted data are presented as means ± SE. Potential confounding variables including maternal pre-pregnancy BMI, age, parity, cigarette smoking, ethnicity and infant gender were controlled when appropriate. A dummy variable was created for ethnicity. Potential confounders were defined as those which altered the adjusted odds ratio or means by at least 10%, and assessed by comparing crude and adjusted data. The statistical significant level was defined as p<0.05. All statistical analyses were performed using SAS v.9.1 (SAS Institute, Inc., Cary, NC).

## Results

Maternal characteristics including age, pre-pregnancy BMI, parity, cigarette smoking and infant gender were not significantly different between cases and controls (Table 1). As expected, cases of spontaneous preterm delivery had significantly shorter gestations at delivery (p<0.0001), lower infant birth weights (p<0.0001) and more were African American (52.17%) than controls (15.79%, p<0.05). In addition, two cases of spontaneous preterm delivery were complicated by preeclampsia (8.7%). The proportion with reduced fetal growth (birth weight <25^th^ percentile for gestational age) was different in cases (n=7, 30.4%) and controls (n=1, 5.2%, p=0.0541 by Fisher’s exact test).

### Selected dietary nutrients intake in cases and controls

We tested dietary nutrient intake between cases and controls. We found that dietary free and total choline intake at entry were significantly lower in preterm delivery cases without reduced fetal growth (p<0.05 vs. cases with reduced fetal growth for free and total choline; p<0.01 vs. controls for total choline) (Table 1). Cases with and/or without reduced fetal growth also had lower folate intake including dietary and dietary plus prenatal supplements as well as lower betaine intake, although the differences were not significant. Dietary macronutrients and total other B vitamins were not different among groups.

### Gene specific methylation

Adjusted mean DNA methylation (%) is shown by case-control status in Table 2 at entry, DNA methylation tended to be lower in the cases but differences were not statistically significant (p>0.05 for each gene, Table 2). During the 3^rd^ trimester cases had lower DNA methylation in all 24 genes, the differences were statistically significant in 15 of 24 genes (62.5%) thus suggesting decreased overall methylation (p<0.045 to p<0.003, Table 3). When results from all 24 genes were combined, the overall mean methylation (%) between cases (mean ± SE, 15.84 ± 1.81) and controls (37.04 ± 8.07) was also highly significant (p<0.01).

**Figure 1:**
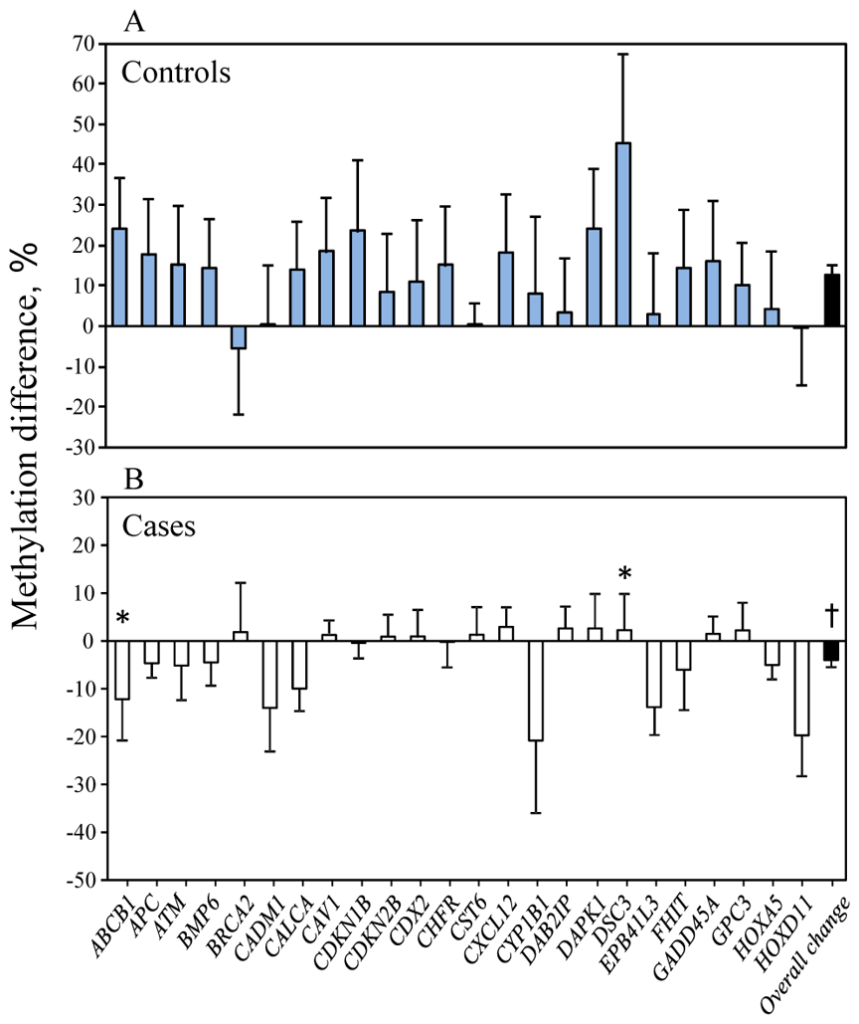
The changes of DNA methylation (%) between entry and the 3rd trimester in spontaneous preterm delivery cases and controls. Models were adjusted for maternal age, pre-pregnancy BMI, ethnicity, parity and cigarette smoking. Shown are mean ± SE. * p<0.05; † p<0.0001. A. Controls showed significantly increased methylation (overall increase in methylation was 12.6 ± 2.19%). B. Cases showed the opposite with decreased methylation compared to the controls (overall decrease in methylation was −4.0 ± 1.5%, p<0.0001).

A different pattern for the change in DNA methylation between entry and 3^rd^ trimester was observed in cases and controls (Figure 1). Cases showed decreased methylation (overall changes in methylation of 24 genes combined was −4.0 ± 1.5% (mean ± SE, ranging from −20.8 to +2.9%, Figure 1B). Whereas controls showed the opposite, with significantly increased methylation (overall changes in methylation was +12.6 ± 2.2%, ranging from −5.4 to +45.3%, p<0.0001 vs. cases, Figure 1A). Significant changes for individual genes were found in 2 genes (*ABCB1* and *DSC3*, p=0.023 and p=0.038, respectively).

### Clonal bisulfite sequencing analysis

**Figure 2:**
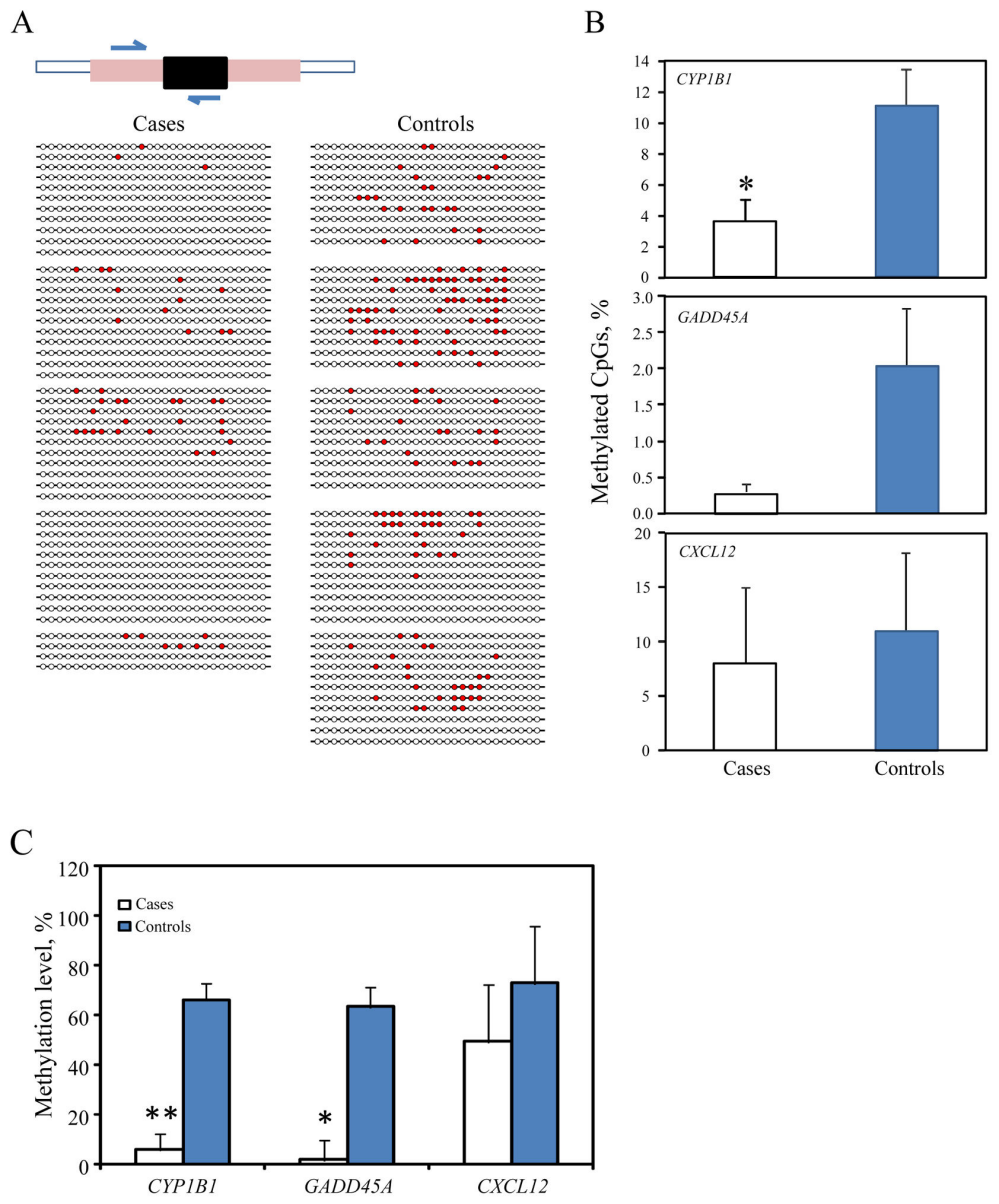
DNA methylation by clonal bisulfite sequencing and by Methyl-Profiler PCR array in a subset of the same sample. Shown are mean ± SE. * p<0.05. **A:** Clonal bisulfite sequencing of the *CYP1B1* gene as an example of 3 genes in cases and controls (n=5 for each): selected genomic regions were analyzed (208-bp, 28 CpG sites, +733/+940 to mRNA 5′ end, 2p22.2, Genbank accession number NM_000104). PCR amplification of MethylScreen shown in black bar within exon 2 (pink bar). Half arrowheads indicate the PCR primer pair used for Na bisulfate modification-cloning-sequencing. Each row represents an individual clone from the post-bisulfite PCR product. Each column represents a CpG site. Filled circles (in red) indicated methylated CpG sites. **B:** Percent of methylated CpG sites in all 3 genes (*CYP1B1, GADD45A* and *CXCL12*) in cases and controls (n=5 for each) by clonal bisulfite sequencing. The methylated % was calculated separately from each clone and the average total number of clones for cases and controls were plotted in the histogram graph panel. **C:** DNA methylation at 3^rd^ trimester by Methyl-Profiler PCR array analysis in the same subjects of 3 genes.

To confirm above observation, we next selected three genes (*CYP1B1, GADD45A* and *CXCL12*) from a subset of the same sample at 3^rd^ trimester (n=5 for each group) and conducted bisulfite sequencing of CpG sites in the same regions examined by the Methyl-Profiler PCR array above using the same subjects. Clonal bisulfite sequencing of *CYP1B1* gene is shown as an example (Figure 2A). Cases of preterm had a decreased percentage of methylated CpG sites in all 3 genes as compared to the controls but only *CYP1B1* methylation was statistically significant (p=0.038, Figure 2B); DNA methylation in these 3 genes (Figure 2C, data shown are extracted from data shown in Table 3) showed that cases had decreased DNA methylation (p<0.05 for *GADD45A,* p<0.01 for *CXCL12*, Figure 2C). Thus, the presence of DNA hypomethylation in cases of preterm delivery was consistent revealed by both technologies of clonal bisulfite sequencing and the Methyl-Profiler PCR Array.

### Reduced fetal growth and DNA demethylation

To identify whether DNA methylation was associated with reduced fetal growth, we divided cases into subgroups of spontaneous preterm delivery - those with and those without reduced fetal growth - comparing them to term controls (Table 4). Owing to longer gestation duration, controls delivered at term had infants with significantly greater birth weight, length, head and chest circumferences as compared to cases with and cases without reduced fetal growth. Only birth weight and length were significantly different between the cases (p<0.05).

A graded pattern of maternal DNA methylation was obtained with the lowest methylation level observed in cases delivering preterm with reduced fetal growth, followed by cases delivering preterm without reduced fetal growth. The highest methylation level was found in controls all of whom delivered AGA infants at term (Table 5). The mean DNA methylation in 15 of 24 genes was significantly different when each subgroup of cases with and without reduced fetal growth was compared to controls (p for trend <0.048 to p<0.003 for each gene, Table 5).

## Discussion

Our findings showed DNA hypomethylation in 15 of 24 tumor-related genes during the 3^rd^ trimester in women with spontaneous preterm delivery, particularly when accompanied by reduced fetal growth. In addition, there was a gradation in DNA hypomethylation among cases -such that cases who delivered preterm with reduced fetal growth had the lowest levels of methylation, cases without reduced fetal growth were next followed by controls - all of whom delivered at term.

The study was prospective; cases with spontaneous preterm delivery and term controls were randomly selected from a large, well characterized cohort studied from entry to care until delivery. Women with a prior history of preterm delivery, the strongest risk factor, were not included; other potential confounders including maternal age, pre-pregnancy BMI, ethnicity, parity, cigarette smoking and infant gender were controlled. Two assays, one from early and the other from later in pregnancy, were conducted blind to the case-control status to minimize systematic bias. The results were confirmed by clonal bisulfite sequencing for 3 of the genes. Thus, the differences detected are novel, and robust as well as independent of many known risk factors and biases.

### Altered DNA methylation and spontaneous preterm delivery

Our finding of an association between 3^rd^ trimester DNA hypomethylation to spontaneous preterm contributes to the limited information on the topic. It is entirely plausible but not yet proven that some behavioral (diet, smoking, stress) and other factors (ethnicity, BMI) that increase risk for preterm delivery do so by altering DNA methylation.

Prior research showed that lower DNA methylation in long interspersed nuclear element-1 (line-1) from maternal white blood cells during the 1^st^ but not the 2^nd^ trimester was associated with shorter gestation duration amounting to 0.45 weeks on average (95% CI 0.12, 0.78) and a 3- to 4-fold increased risk of preterm birth [16]. Several studies analyzed DNA methylation in cord blood collected at delivery to link with preterm delivery and fetal growth, with mixed results [9,14–15]. Parets et al identified 29 CpG sites that were associated with preterm birth <34 weeks in fetal leukocyte DNA from African American gravidae. Mean methylation was decreased in 19 CpG sites (66%); the methylation in other 10 CpG (34%) sites was increased compared to term births [14]. In contrast, Liu et al reported cord blood DNA methylation measured by pyrosequencing did not differ in any of 9 genes from differential methylation regions (DMR) when women with a spontaneous preterm delivery, a prior history of preterm delivery or a medically indicated preterm delivery were compared to one another [9]. This study, however, did not include term deliveries as a comparison group.

### DNA hypomethylation and reduced fetal growth

Our preliminary observations suggest that epigenetic alternations, as indicated by DNA hypomethylation, could be associated with fetal growth and development. SGA and reduced fetal growth are common in preterm delivery, both are associated with perinatal and childhood morbidity and have lifelong consequences for increased cardiovascular risk in both mother and offspring [1,2]. Our data thus imply that hypomethylation of maternal DNA during gestation could be more pronounced with severely restricted fetal growth but this is speculative on our part.

Maternal nutritional status at conception and during pregnancy regulates DNA methylation and influences fetal growth and development in animal models [32]. In human studies, decreased cord blood DNA methylation of *MEG2* and *IGF2* was observed in low birth weight infants and mediated by maternal depressed mood [33]. Cord blood DNA methylation showed some association with body composition in childhood [19]. However, Tobi et al found no difference in DNA methylation in several genetic loci including IGF2 in individuals who were born preterm with and without SGA [15]. Likewise, risk factors related to SGA (preeclampsia and smoking) also did not differ in methylation status [15]. Although these data are not from maternal samples, they do suggest that DNA methylation potentially influences fetal growth and that epigenetic changes other than methylation may be involved in fetal and childhood growth.

DNA methylation, an epigenetic modification to the genome, can influence gene transcription, genomic stability and the regulation of other cellular processes [8,12,34]. Altered DNA methylation or mRNA expression of tumor-related genes occurs in many non-tumor states [21,33,35,36]. Previous studies have linked the function of tumor-related genes with pregnancy and fetal development (see S1 table). For example, hypermethylation of the adenomatous polyposis coli (*APC* gene promoter (a tumor suppressor gene) in human term placenta suggests that silencing of tumor suppressor genes is an integral part of normal placental development [35].

The physiological demands of pregnancy are a metabolic and cardiovascular ‘stress test’ [1,37]. Women who fail the test may be predisposed to adverse pregnancy outcome such as preterm delivery [5,37]. We have previously reported that poor maternal nutritional status, imbalanced metabolism, increased oxidative stress and/or an exacerbated inflammatory response all are associated with a number of adverse pregnancy outcomes including preterm delivery and SGA [38–41]. Choline is an essential nutrient [42–47]. Both human studies and animal models suggest that choline intake during pregnancy has the potential to modify epigenetic states in the offspring with implications for adult health and later chronic disease risk [48–52]. We observed that dietary free and total choline intakes at entry were significantly lower in cases of preterm delivery (Table 1). This work provides evidence for the need to examine the extent to which dietary nutrients also affect DNA methylation. Although none of the women delivered infants traditionally described as SGA (<10^th^ percentile), our data suggest that it would be important to examine maternal hypomethylation in such cases. Thus, we hypothesize that epigenetic modification is a maternal response to a dramatically changed environment. A poor adaptation or incomplete compensation can induce DNA hypomethylation and other alterations which increase susceptibility to preterm delivery, particularly with reduced fetal growth.

We acknowledge some limitations. DNA methylation varies with cell or tissue type and peripheral blood cell composition can as well affect levels of DNA methylation [53–58]. We used maternal peripheral blood samples for DNA methylation without the adjustment for the proportion of white cell types. However, for the past decades, whole blood cells (buffy coat) have been widely used for research because they are easily accessible and provide the greatest amount of DNA for analysis. In our study, samples were obtained prospectively from generally healthy women, starting in early pregnancy, and collected in the same manner before the women delivered preterm (cases) or at term (controls). Several factors reported to be associated with DNA methylation including age, BMI, race and smoking status were controlled in our analysis [59–63] thus potentially reducing variation from many known factors other than white cell type.

In addition, we used a standard that adjusts for maternal ethnicity and other factors related to fetal growth restriction. Using a standard that adjusted for ethnicity is controversial given that environmental or behavior exposures more common in certain ethnic groups might be causing poor fetal growth [64–67]. This topic should be addressed in future epigenetic research.

## Conclusion

Our finding support the idea that epigenetic dysregulation may be one of underlying mechanisms for spontaneous preterm delivery particularly with reduced fetal growth. These data are potential significant for the identification of women at risk of preterm delivery. More importantly, because DNA methylation is modifiable and potentially reversible, the identification of modifiable factors like diet that modulate or influence epigenetic regulation holds promise for new insights into the prevention of this critical problem.

## Supplementary Material

Suppl table

## Figures and Tables

**Table 1:** Maternal diet and other characteristics for cases of spontaneous preterm delivery and term controls*.

	All preterm cases	Preterm cases and reduced fetal growth	Preterm cases and AGA	Term controls and AGA
n	23	7	16	19
Age (yr.)	21.5 ± 1.0	21.6 ± 1.9	21.9 ± 1.4	22.2 ± 1.3
BMI (kg/m^2^)	26.4 ± 1.4	22.3 ± 1.8	28.0 ± 1.3	25.7 ± 1.2
Nullipara	7 (30.43)	3 (42.86)	4 (25.00)	8 (42.11)
Cigarette smoking	4 (17.39)	1 (14.29)	3 (18.75)	2 (10.53)
Ethnicity				
Hispanic	9 (39.13)	3 (37.50)	6 (37.50)	11 (57.89)
African American	12 (52.17)	3 (37.50)	9 (56.25)	3 (15.79)
Caucasian	2 (8.70)	1 (14.29)	1 (6.25)	5 (26.32)
Preeclampsia	2 (8.69)	2 (28.57)	0	0
Gestational age at delivery (weeks)	34.3 ± 0.4	34.6 ± 0.7	34.5 ± 0.7	39.2 ± 0.3†‡
Infant birth weight (g)	2609 ± 170	2137 ± 225	2866 ± 159	3605 ± 150†‡
Infant gender (male)	9 (39.13)	1 (14.29)	8 (50.00)	9 (47.36)
Reduced fetal growth				
<10^th^ percentile for gestation	0	0	0	0
<25^th^ percentile for gestation	7 (30.43)	7 (100)	0	1 (5.26)
Dietary nutrients intake at entry (per day)				
Total Fat (g)	81.7 ± 5.0	89.4 ± 8.4	77.5 ± 6.0	77.0 ± 6.3
Protein (g)	87.3 ± 6.7	81.9 ± 11.2	89.5 ± 8.1	86.0 ± 8.4
Carbohydrate (g)	290.7 ± 16.3	279.9 ± 27.5	297.5 ± 19.8	301.1 ± 20.7
Free choline (mg)	52.5 ± 5.4	63.9 ± 6.1	48.7 ± 4.4§	57.9 ± 4.6
Total choline (mg)	314.0 ± 32.0	404.1 ± 49.6	262.0 ± 35.7§¶	408.9 ± 37.3
Betaine (mg)	132.8 ± 17.6	106.5 ± 31.5	145.5 ± 21.3	131.8 ± 22.3
Total other B Vitamins (mg)	10.3 ± 1.7	9.5 ± 2.8	10.5 ± 2.0	8.3 ± 2.1
Folate (μg)	300.5 ± 50.7	308.7 ± 60.3	288.4 ± 43.4	327.7 ± 45.3
Folate (supplement, μg)	238.6 ± 96.8	339.7 ± 167.3	238.1 ± 120.5	479.4 ± 125.7
Total folate (diet and supplement, μg)	539.2 ± 106.2	648.4 ± 182.2	526.5 ± 131.3	807.1 ± 136.9

* Data are means ± SE or n (%). Reduced fetal growth for gestation was defined as infant birth weight <25^th^ percentile for gestational age, using a standard which adjusts for parity, infant gender and ethnicity. AGA, infant birth weight was appropriate for gestational age. Data for dietary nutrient intakes were adjusted for total energy intake. Total other B vitamins include vitamins B2, B6 and B12.

† p<0.0001 vs. preterm cases with reduced fetal growth;

‡ p<0.0001 vs. preterm cases and AGA;

§ p<0.05 vs. preterm cases with reduced fetal growth (ranges of p was 0.02 to 0.04).

¶ p<0.01 vs. term controls and AGA (p =0.002).

**Table 2:** Comparison of DNA methylation (%) between spontaneous preterm cases and term controls at entry to care*.

Gene symbol	Cases (n=23)	Controls (n=19)	p-value
*ABCB1*	16.98 ± 6.14	16.25 ± 6.96	0.938
*APC*	5.56 ± 4.67	11.47 ± 5.15	0.407
*ATM*	12.49 ± 5.64	13.30 ± 6.40	0.926
*BMP6*	9.58 ± 5.58	19.12 ± 6.15	0.264
*CAV1*	3.28 ± 4.31	11.07 ± 4.89	0.246
*CADM1*	18.30 ± 7.02	26.15 ± 7.96	0.470
*CDKN1B*	6.09 ± 4.72	12.84 ± 5.35	0.356
*CDKN2B*	6.59 ± 6.20	20.69 ± 6.84	0.140
*CHFR*	5.19 ± 5.48	20.24 ± 6.04	0.077
*CST6*	77.00 ± 3.58	83.47 ± 3.94	0.238
*DAB2IP*	5.01 ± 4.95	20.51 ± 5.46	0.045
*DAPK1*	13.39 ± 5.84	19.35 ± 6.44	0.502
*DSC3*	8.01 ± 6.83	12.53 ± 7.51	0.670
*EPB41L3*	16.10 ± 6.42	20.43 ± 7.12	0.658
*FHIT*	11.91 ± 6.57	19.60 ± 7.09	0.438
*GADD45A*	8.03 ± 5.58	18.46 ± 6.15	0.222
*GPC3*	45.96 ± 4.77	49.35 ± 5.26	0.640
*HOXA5*	7.77 ± 5.61	20.67 ± 6.19	0.136
*HOXD11*	39.30 ± 8.04	30.62 ± 8.66	0.472
*BRCA2*	30.21 ± 8.09	43.66 ± 8.77	0.277
*CALCA*	30.06 ± 4.81	26.42 ± 5.32	0.618
*CDX2*	13.88 ± 5.37	17.53 ± 6.09	0.660
*CXCL12*	8.56 ± 5.44	21.53 ± 5.97	0.122
*CYP1B1*	61.33 ± 6.82	59.53 ± 6.82	0.858

* Data are means ± SE.

Models were adjusted for age, BMI, parity, ethnicity and cigarette smoking.

**Table 3:** Comparison of DNA methylation (%) between spontaneous preterm delivery cases and controls during the 3^rd^ trimester*.

Gene symbol	Cases (n=23)	Controls (n=19)	p-value
*ABCB1*	6.31 ± 7.84	33.85 ± 8.27	0.024
*APC*	0.63 ± 6.41	30.27 ± 7.11	0.005
*ATM*	6.85 ± 6.92	28.70 ± 7.67	0.045
*BMP6*	4.95 ± 5.87	33.45 ± 6.47	0.003
*CAV1*	4.78 ± 6.35	27.68 ± 7.00	0.022
*CADM1*	5.63 ± 6.10	25.60 ± 6.61	0.064
*CDKN1B*	5.98 ± 6.96	36.65 ± 8.26	0.009
*CDKN2B*	9.08 ± 7.20	27.28 ± 7.94	0.103
*CHFR*	5.56 ± 6.93	34.52 ± 7.65	0.009
*CST6*	77.36 ± 4.11	84.63 ± 4.66	0.253
*DAB2IP*	8.44 ± 6.75	23.12 ± 7.27	0.154
*DAPK1*	13.12 ± 8.02	46.57 ± 8.02	0.007
*DSC3*	12.35 ± 8.67	25.86 ± 9.76	0.327
*EPB41L3*	2.61 ± 5.66	23.03 ± 6.41	0.024
*FHIT*	7.39 ± 7.42	31.28 ± 8.00	0.037
*GADD45A*	10.13 ± 7.35	34.00 ± 7.93	0.036
*GPC3*	47.62 ± 5.35	61.83 ± 5.77	0.084
*HOXA5*	2.32 ± 5.90	25.31 ± 6.36	0.013
*HOXD11*	19.17 ± 7.65	33.54 ± 8.96	0.243
*BRCA2*	32.96 ± 8.40	31.99 ± 9.45	0.941
*CALCA*	20.00 ± 5.56	39.76 ± 6.17	0.025
*CDX2*	14.28 ± 7.41	29.28 ± 8.20	0.187
*CXCL12*	11.53 ± 7.52	40.15 ± 8.11	0.016
*CYP1B1*	45.53 ± 9.67	76.05 ± 10.09	0.036

* Data are means ± SE.

Models were adjusted for age, BMI, parity, ethnicity and cigarette smoking.

**Table 4:** Infant birth weight and size: reduced fetal growth and spontaneous preterm delivery*.

	Group 1 (Preterm cases and reduced fetal growth)	Group 2 (Preterm cases and AGA)	Group 3 (Term controls and AGA)	P for trend
n	7	16	18	
Birth length (cm)	44.32 ± 1.41	47.53 ± 0.98†	51.78 ± 0.90** †	0.0001
Head circumference (cm)	28.83 ± 1.66	31.49 ± 1.15	33.56 ± 1.05‡ §	0.049
Chest circumference (cm)	28.62 ± 1.17	30.84 ± 0.84	33.07 ± 0.75†	0.005
Birth weight (g)	2168 ± 248	2840 ± 171§	3614 ± 157** ¶	<0.0001
Gestational age at delivery (week)	34.30 ± 0.72	34.54 ± 0.50	39.28 ± 0.46** ¶	<0.0001

* Data are mean ± SE. Reduced fetal growth for gestation was defined as infant birth weight <25^th^ percentile for gestational age using a standard which adjusts for parity, infant gender and ethnicity.

AGA, infant birth weight was appropriate for gestational age. One term control with reduced fetal growth was excluded from the analysis.

Models were also adjusted for age, BMI and smoking

** p≤0.0001 vs. group 1

† p<0.01 vs. group 2 (ranges of p was 0.002 to 0.006)

‡ p=0.04 vs. group 2

§ p<0.05 vs. group 1 (ranges of p was 0.032 to 0.046).

¶ p<0.0001 vs. group 2

**Table 5:** Comparison of 3^rd^ trimester DNA methylation (%) among cases of spontaneous preterm delivery (with and without reduced fetal growth) and term controls*.

Gene symbol	Group 1 (Preterm cases and reduced fetal growth)	Group 2 (Preterm cases and AGA)	Group 3 (Term controls and AGA)	P for trend
**n**	7	16	18	
*ABCB1*	6.11 ± 13.92	5.66 ± 9.11	36.38 ± 8.12**	0.021
*APC*	0.38 ± 11.13	0.92 ± 7.60	31.90 ± 7.13†‡	0.005
*ATM*	1.05 ± 12.42	11.23 ± 8.49	28.88 ± 7.97†	0.023
*BMP6*	0.32 ± 11.58	6.54 ± 7.66	35.67 ± 7.22** †	0.003
*CAV1*	0.40 ± 12.06	5.61 ± 7.94	30.13 ± 7.31** †	0.015
*CADM1*	6.79 ± 11.50	6.71 ± 7.83	25.32 ± 7.20	0.096
*CDKN1B*	5.68 ±12.53	5.81 ± 8.67	39.52 ± 8.44†‡	0.012
*CDKN2B*	0.04 ± 12.68	11.79 ± 8.38	29.90 ± 7.90†	0.028
*CHFR*	0.01 ± 12.58	7.37 ± 8.32	36.99 ± 7.85** §	0.004
*CST6*	71.16 ± 7.74	79.94 ± 5.07	84.63 ± 4.81	0.147
*DAB2IP*	5.75 ± 13.39	8.28 ± 8.18	25.44 ± 7.57	0.110
*DAPK1*	12.23 ± 13.99	13.53 ± 10.50	49.23 ± 8.52‡†	0.011
*DSC3*	22.37 ± 15.65	7.78 ± 10.72	27.72 ± 10.01	0.521
*EPB41L3*	1.38 ± 10.69	4.01 ± 7.06	23.53 ± 6.86** †	0.024
*FHIT*	0.06 ± 12.76	10.42 ± 8.72	33.32 ± 7.95†	0.018
*GADD45A*	5.30 ± 14.39	11.36 ± 8.79	36.39 ± 8.14**	0.025
*GPC3*	45.13 ± 9.78	47.94 ± 6.67	63.06 ± 5.95	0.067
*HOXA5*	4.26 ± 11.04	1.25 ± 7.53	26.80 ± 6.72‡	0.029
*HOXD11*	24.02 ± 14.31	15.78 ± 9.51	36.89 ± 9.36	0.287
*BRCA2*	24.20 ± 15.49	35.98 ± 10.09	31.17 ± 9.50	0.837
*CALCA*	17.42 ± 10.28	21.35 ± 6.99	41.20 ± 6.43**	0.026
*CDX2*	11.91 ± 13.60	14.39 ± 9.26	31.85 ± 8.42	0.135
*CXCL12*	15.88 ± 13.67	7.88 ± 9.33	43.72 ± 8.33‡	0.025
*CYP1B1*	38.58 ± 14.73	53.16 ± 13.38	75.07 ± 10.29†	0.048

* Data are means ± SE. Reduced fetal growth for gestation was defined as infant birth weight <25th percentile for gestational age using a standard which adjusts for parity, infant gender and ethnicity. AGA, infant birth weight was appropriate for gestational age. One term control with reduced fetal growth was excluded from the analysis.

Models were also adjusted for age, BMI and cigarette smoking.

** p<0.05 vs. group 2 (ranges of p was 0.028 to 0.045)

† p<0.05 vs. group 1 (ranges of p was 0.018 to 0.043)

‡ p<0.01 vs. group 2 (ranges of p was 0.01 to 0.007)

§ p<0.01 vs. group 1 (ranges of p was 0.01 to 0.008)
