# Supplementary material for: Spontaneous Preterm Delivery, Particularly with Reduced Fetal Growth, is Associated with DNA Hypomethylation of Tumor Related Genes
Source: J Pregnancy Child Health. Author manuscript; Available in PMC 2016 Aug 5. (PMC4975560; doi:10.4172/2376-127X.1000215)
Supplement: Suppl table [file NIHMS805682-supplement-Suppl_table.pdf]

Supporting Information

S1 Table. Information of 24 tumor-related genes

Gene symbol*	**Gene name***	**Chrom. Location***	**Function related to pregnancy/development**	**EpiTect Methyl cat # (Qiagen)**
ABCB1	ATP-binding cassette, sub-family B (MDR/TAP), member 1	7q21.12	Fetoplacental protection in animals [36]	MePH28467-1A
APC	Adenomatous polyposis coli	5q21-q22	Human placental development [35]	MePH28469-1A
ATM	ATM serine/threonine kinase	11q22-q23	Intrauterine growth restriction in animals [47]	MePH28470-1A
BMP6	Bone morphogenetic protein 6	6p24-p23	Gestational age in humans[48]	MePH28471-1A
CADM1	Cell adhesion molecule 1	11q23.2	Fetal pancreatic islet cells function in animals [49]	MePH28474-1A
CAV1	Caveolin 1, caveolae protein	7q31.1	Preeclampsia [50]	MePH28476-1A
CDKN1B	Cyclin-dependent kinase inhibitor 1B	12p13.1-p12	Organ growth in *vitro* [51]	MePH28481-1A
CDKN2B	Cyclin-dependent kinase inhibitor 2B	9p21	Gestational diabetes in humans [52]	MePH28484-1A
CHFR	Checkpoint with forkhead and ring finger domains, E3 ubiquitin protein ligase	12q24.33	Gestational choriocarcinoma [53]	MePH28486-1A
CST6	Cystatin E/M	11q13	Establishment and maintenance of pregnancy in animals [54]	MePH28487-1A
DAB2IP	DAB2 interacting protein	9q33.1-q33.3	Human fetal development [55]	MePH28490-1A
DAPK1	Death-associated protein kinase 1	9q21.33	Preeclampsia in humans [56]	MePH28491-1A

DSC3	Desmocollin 3	18q12.1	Embryonic development in animals [57]	MePH28492-1A
EPB41L3	Erythrocyte membrane protein band 4.1-like 3	18p11.32	Unknown	MePH28493-1A
FHIT	Fragile histidine triad	3p14.2	Fetal development in animals [58]	MePH28495-1A
GADD45 A	Growth arrest and DNA-damage-inducible, alpha	1p31.2	Preeclampsia [18]	MePH28496-1A
GPC3	Glypican 3	Xq26.1	Human fetal growth [59]	MePH28497-1A
HOXA5	Homeobox A5	7p15.2	Human fetal growth and development [60]	MePH10012-1A
HOXD11	Homeobox D11	2q31.1	Fetal bone formation in animals [61]	MePH28501-1A
BRCA2	Breast cancer 2	13q12.3	Pregnancy increased risk of breast cancer in carriers of BRCA2 [62]	MePH28473-1A
CALCA	Calcitonin-related polypeptide, alpha	11p15.2	Human placental development and preeclampsia [63]	MePH28475-1A
CDX2	Caudal type homeobox 2	13q12.3	Trophoblast cell invasion in *vitro* [64]	MePH28485-1A
CXCL12	Chemokine (C-X-C motif) ligand 12	10q11.1	Trophoblast cell proliferation or differentiation in animals and humans [65-66]	MePH28488-1A
CYP1B1	Cytochrome P450, family 1, subfamily B, polypeptide 1	2p22.2	Primary congenital glaucoma [67]	MePH28489-1A

* Information was obtained from Gene database (www.ncbi.nih.gov) and TSGene (http://bioinfo.mc.vanderbilt.edu/TSGene)
